# Persistent Inflammation and Nitric Oxide Dysregulation Are Transcriptomic Blueprints of Subglottic Stenosis

**DOI:** 10.3389/fimmu.2021.748533

**Published:** 2021-12-20

**Authors:** Hoang C. B. Nguyen, Tiffany N. Chao, Noam A. Cohen, Natasha Mirza

**Affiliations:** ^1^Perelman School of Medicine, University of Pennsylvania, Philadelphia, PA, United States; ^2^Department of Otorhinolaryngology-Head and Neck Surgery, University of Pennsylvania, Philadelphia, PA, United States; ^3^Division of Otolaryngology, Philadelphia Veterans Affairs Medical Center, Philadelphia, PA, United States

**Keywords:** subglottic stenosis, mucosal swab, transcriptomic profiling, granulomatous disease, nitric oxide

## Abstract

Subglottic stenosis (SGS) is a recurrent, obstructive, fibroinflammatory disease of the upper airway resulting in severe dyspnea, dysphonia, as well as other potentially fatal complications. Although aberrant inflammation and wound-healing are commonly associated with pathogenesis, the mechanism through which such processes occur and recur in affected patients remains poorly studied. Here we report that transcriptomic profiling of laryngotracheal regions from minimally-invasive mucosal swabs of SGS patients reveals a distinctively pro-inflammatory gene signature. Surprisingly, comparative genomics between SGS patients and mice with direct laryngotracheal injury suggest that SGS patients bear more resemblance to the acute than chronic phase of injury. Furthermore, functional and regulatory network analyses identify neutrophilic involvement through hyper-activation of NF-κB and its downstream inflammasome as a potential master regulator. Interestingly, nitric oxide synthesis was found to be downregulated in SGS patients compared to healthy controls. Thus, SGS represents a state of immunodeficiency whereby defective immune clearance triggers recurrent, long-lasting production of pro-inflammatory cytokines.

## Introduction

Subglottic stenosis (SGS) is a disease of pathological constriction of the upper airway below the level of the vocal cord that symptomatically manifests as fixed extrathoracic blockage of pulmonary ventilation that could potentially be life-threatening. Though airway trauma from prolonged endotracheal intubation is the most common cause of SGS (up to 70% of case), a significant portion of patients (19%) also present with underlying autoimmune disease or no apparent causes (idiopathic) (1). Regardless of the history, SGS patients are believed to share common features of airway epithelial injury, with excessive inflammation and hypertrophic scar formation leading to obstruction of airway lumen (2). Surgery remains the standard of care for SGS patients, including endoscopic procedures such as balloon serial dilation, with or without laser resection, as well as open surgery which is often associated with more significant risks though potentially a more durable long-term cure. Unfortunately, SGS often recurs requiring repeated surgical interventions (3).

Over the past decade, researchers and clinicians have started to shed light on the pathogenesis of SGS. In addition to histological evidence of exuberant granulation tissue and accumulation of permanent scar tissue, prior studies have shown an increased in several markers of inflammation and fibrosis, such as Interleukin (IL)-1, transforming growth factor beta (TGF-β), platelet-derived growth factor (PDGF), Interferon gamma (IFNG), as well as prostaglandin (PG)-2 from excised stenotic tissues (4–8). The importance of the immune system in the development of SGS was further emphasized by findings that immunodeficient mice failed to develop SGS (9), and that human subglottic tissues were significantly enriched for CD8+ resident memory T cells (10) as well as CD4+ T cells with increased expression of PD-1 and PD-L1 (11, 12). Interestingly, even though SGS is strongly linked to inflammation, the efficacy of steroid treatment for SGS remains variable and primarily anecdotal in the literature (13, 14).

The scarcity of definitive treatment for SGS could be attributed to significant knowledge gaps in pathophysiology as well as a paucity of insights into the natural evolution of the disorder. Such challenges in turn stem from a lack of not only minimally invasive, systemic methods of studying human laryngotracheal regions, but also effective animal model until very recently (9). As a result, most of our current understanding has been from piece-meal, heavily-targeted investigations into different components of such a complex disease.

In this study, we sought to overcome limitations from previous studies as well as better elucidate the molecular mechanisms of SGS pathogenesis *via* utilizing next-generation sequencing to unbiasedly examine the transcriptomic alterations from airway epithelial injury. Combining unbiased transcriptomic studies from both minimally-invasive mucosal swabs of SGS patients and murine tracheas after direct laryngotracheal injury, here we identify a persistent hyper-inflammatory trademark of SGS airway that is associated with down-regulation of nitric oxide synthesis.

## Materials and Methods

### Statistics and Reproducibility

All data were presented with individual biological replicates shown when appropriate. RStudio (v1.0.153) with R-packages: DESeq2(v3.13), ggplot2 (v3.3.0), latticeExtra(v0.6-29), pheatmap (v1.0.12) softwares and Graphpad Prism (v8.0) were used for graphing and statistical analysis. All statistical tests are fully described in the figure legends. In general, *p* values were calculated using two-tailed Student’s t-test for pairwise comparisons and one-way ANOVA for multiple comparisons. Mann-Whitney U-test was used to compare the difference in distributions of independent groups with no assumption of normal distribution. Statistics reported by Enrichr (q-values) were adjusted by Benjamini-Hochberg (BH) method. Correlations with associated p values from two-sided t-tests were calculated by R function *cor()*.

### Human Studies

Mucosal swab specimens from stenotic tracheas were taken from 11 patients with symptomatic SGS. Patients with SGS secondary to endotracheal intubation, granulomatosis with polyangiitis, tracheostomy, as well as idiopathic were included in this study. On average, patients’ endotracheal extubation or previous surgery for SGS had occurred 3 months before their tissue biopsies (range, 6 weeks to 4 months). Controls were collected from patients without subglottic lesions. Sample collections were performed in the operating room under general anesthesia. All patients signed informed consent.

### Animal Studies

All animal studies were approved by the Corporal Michael J. Crescenz Philadelphia Veterans Affairs Medical Center Institutional Animal Care and Use Committee. All C57BL/6 mice were housed in a temperature-controlled specific-pathogen-free facility under 12-hour light/dark cycles. Laryngotracheal complex (LTC) transfer was done as previously described (8). Briefly, LTC transplants were performed using donor tracheal segments from 8-week-old, 20- to 25-g female C57BL/6 mice (Jackson Laboratories, Bar Harbor, Maine). LTCs were harvested and divided into 2 groups: 1-uninjured, and 2-mechanically injured using a wire brush. One donor LTC from each group was placed in deep dorsal subcutaneous pockets of C57BL/6-recipient mice, for a total of 3 transplanted tracheas per recipient mouse. Immediately after, or after 1, 3, and 4 weeks, the transplanted LTCs were harvested from both C57BL/6 recipient mice.

### RNA Isolation

Human laryngotracheal regions were directly visualized with microdirect laryngoscope and mucosal swabs were collected with sterile 1.27 x 0.5cm surgical patties. Each site was lightly brushed for a few seconds, and the patties were subsequently placed in a vial 5ml conical tube containing RNAlater immediately after collection from the operating room. The tubes were vortex-mixed for 30 seconds, patties were removed, and the RNAlater solution decanted following 15 minutes of centrifugation at 16,000 x g. This step ensured maximum recovery of epithelial cells. The decanted RNAlater was collected in a fresh tube and centrifuged at 2,000 x g for 5 minutes. Pellets were resuspended in 500 μL of TRIzol. Samples were incubated for 1 hour at room temperature, before lysing the cells by repeated passage through a 29-G syringe. We then proceeded with the TRIzol RNA extraction protocol as suggested by the manufacturer’s instructions.

Mouse tracheas were homogenized manually by Pellet-Pestle (Kimble, 749515) for 5 minutes with 1mL TRIzol. Samples were then centrifuged at 4°C for 10 minutes at 15,000 x g. In a new tube, 200µL chloroform was added to homogenate and mixed thoroughly before repeating the previous centrifugation. The aqueous chloroform phases were pipetted into new tubes, followed by addition of one volume of 70% EtOH to each sample. Total RNA was purified and collected from RNeasy Plus Mini spin columns according to manufacturer’s instructions (Qiagen, 74804). All RNA was treated with RNase-free DNase (Qiagen) to remove DNA contaminant prior to sequencing.

### Western Blot

Human laryngotracheal swabs were obtained as described previously with the exception that the samples were directly placed in tubes containing ice-cold RIPA buffer (Thermo fisher) with protease and phosphatase inhibitors (Roche). The samples were vortexed vigorously before removal of cellular debris and surgical patties by ultracentrifugation at 21,000 × g for 20 minutes at 4°C. Immunoblot PVDF membranes were scanned using LICOR Odyssey SA Infrared imaging system according to manufacturer’s instructions using anti-iNOS antibody (Abcam ab178945, 1:500 dilution), anti-GAPDH antibody (Abcam ab8245, 1:1000 dilution).

### Gene Expression Analysis (qRT-PCR)

Complementary DNA (cDNA) was made from purified RNA by High Capacity cDNA Reverse Transcription kit (Applied Biosystems, 43-688-14). cDNA was used for quantitative PCR with Power SYBR Green PCR Master Mix (Applied Biosystems) on the QuantStudio 7 Flex Real-Time PCR software and system. Results were analyzed by standard curve and normalized to *36bp4* expression. All primer sequences can be found in **Table 1**.

**Table 1:** qPCR primer sequence.

qPCR primers	Forward (5’-3’)	Reverse (5’-3’)
*NFKBIA*	CTCCGAGACTTTCGAGGAAATAC	GCCATTGTAGTTGGTAGCCTTCA
*SLC31A1*	ATGGAACCATCCTTATGGAGACA	GGAAGTAGCTTATGACCACCTGG
*HIF1A*	GAACGTCGAAAAGAAAAGTCTCG	CCTTATCAAGATGCGAACTCACA
*IL1A*	AGATGCCTGAGATACCCAAAACC	CCAAGCACACCCAGTAGTCT
*IL1B*	ATGATGGCTTATTACAGTGGCAA	GTCGGAGATTCGTAGCTGGA
*CXCR6*	TCCTGGTGAACCTACCCCTG	AAACACCCATTCATGGATGCC
*NFKB1*	AACAGAGAGGATTTCGTTTCCG	TTTGACCTGAGGGTAAGACTTCT
*CCL17*	GACGACAGAAGGGTACGGC	GCATCTGAAGTGACCTCATGGTA
*CD55*	AGAGTTCTGCAATCGTAGCTGC	CACAACAGTACCGACTGGAAAAT

### RNA-Seq Library Preparation and Data Analysis

For RNA-seq, RNA integrity was examined using Agilent High Sensitivity RNA ScreenTape. 100ng-1μg of RNA samples with RNA integrity number >7 were used for RNA cleanup and library preparation with NEBNext^®^ Ultra™ II RNA Library Prep Kit for Illumina^®^ according to manufacturer’s instructions. All barcoded libraries were quantified by KAPA Library Quantification Kit (Roche), and equimolarly pooled for subsequent sequencing. All RNA-seq experiments were done with at least 3 biological replicates per condition. High-throughput sequencing data was generated by Novogene. Sequencing reads were aligned to UCSC hg38 (human) or mm10 (mouse) genome using STAR v 2.6 (15). Read counts were then obtained with featureCounts per standard manual’s instructions (16). Raw counts were normalized using transcript-per-million (TPM) transformation to correct for variations in sequencing depth. Cut-off for minimal expression of any particular gene was set to TPM > 0.1. Variance stabilizing transformation (VST) was performed on normalized counts to correct for variance and dispersion across the means of multiple samples. To assess the overall similarity between samples, we calculated the Euclidean distance between samples using R function *dist* on the VST values. Heatmaps of sample-to-sample distances were generated with R package ‘pheatmap’. Diagonal cells from top left to bottom right indicate 100% similarity (smallest distance, darkest blue) as they are showing the distance of each respective sample to itself. PCA analysis was also performed on VST values. Differentially expressed genes (cutoff defined as FDR < 0.05, > 0.1 TPM were identified using DESeq2 (17). Heat-maps were generated in R with package ‘pheatmap’ by 1) identifying differentially expressed genes with the aforementioned cut-offs and 2) mapping their corresponding z-transformed TPM or log_2_FC values across all biological replicates and treatment conditions. Heat-map color scheme was scaled accordingly for optimal graphical illustrations. Ecdf (empirical cumulative distribution function) from R package latticeExtra was used to visualize the cumulative distribution in terms of log_2_FC of differentially expressed genes identified from human dataset compared to 4 different mouse datasets based on same set of aforementioned genes. Kolmogorov–Smirnov (K-S) test D statistics were employed to measure the degree of global shift of pairwise comparisons (magnitude of the difference in distribution). Gene ontology analyses and transcriptional factor enrichment were performed by Enrichr (18) and TRRUST database (19). Gene set enrichment analyses were performed with GSEA software (20). Transcription factor binding analysis (TBA) was performed with default parameters (https://github.com/jenhantao/tba) with multi-threading and option -p to test for significance with a likelihood ratio test across five independent train-test iterations (21).

### Nitric Oxide Measurement

Nitric oxide (NO) levels from healthy controls and SGS patients swab samples were measured with Nitric Oxide Assay kit (Invitrogen EMSNO) according to manufacturer’s instructions. Chemiluminescence was measured at wavelength 540nm by Luminex 200 and NO concentrations were quantitated using a seven-point standard curve. Briefly, nitrate reductase was used to convert nitrate to nitrite. Nitrite is then detected as a colored azo dye product of the Griess reaction that absorbs visible light at 540 nm. The interaction of nitric oxide in a system is measured by the determination of both nitrate and nitrite concentrations in the samples.

## Results

### Transcriptomic Profiling of Human Laryngotracheal Mucosal Swabs

**Figure 1:**
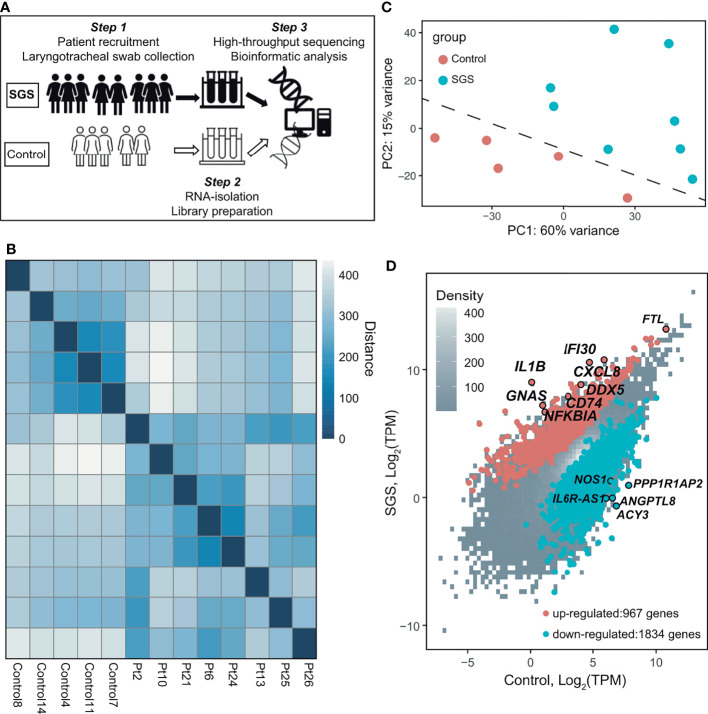
Transcriptomic profiling of human laryngotracheal mucosal swabs. **(A)** Schematics of the experimental design for the collection and processing of laryngotracheal swabs from human controls and SGS patients. **(B)** Heatmap of sample-to-sample distances using the variance stabilizing transformed values from RNA-seq experiments in 5 healthy control and 8 SGS patients (Pt). **(C)** Principle component analysis (PCA) plot using the variance stabilizing transformed values from RNA-seq experiments in 5 healthy control and 8 SGS patients. Dashed line was added as visual aid. **(D)** Scatter plot of RNA-seq data showing differentially expressed genes (absolute fold-change > 2, false discovery rate (FDR) < 0.05, transcripts-per-kilobase million (TPM) > 0.1, up (red) or down (blue) in 5 healthy controls and 8 SGS patients.

We first determined the global gene expression of laryngotracheal regions (LTR) from mucosal swabs of 8 patients with confirmed SGS and 5 healthy controls (**Figure 1A**). Consistent with previous reports (1, 4, 5), all of our SGS patients were Caucasian female in their 50s-60s with recurrent disease that requires multiple rounds of surgical intervention (**Table 2**). Sufficient amount of high-quality RNA was successfully obtained from these specimens to generate sequencing library with high phred score and specificity to the human genome (**Figure S1**). Unbiased hierarchical clustering revealed that the transcriptomic landscapes of SGS patients were more similar to one another (**Figure 1B**), as indicated by the smaller “distance” among SGS samples (darker shades of blue), and visually distinguishable from control samples, as indicated by the larger “distance” (lighter shades of blue to white) when cross-comparing SGS versus healthy samples. Principal component analyses (PCA) further corroborated this distinction between the two groups (**Figure 1C**), suggesting that substantial transcriptional alteration had occurred even at the mucosal layer of LTR. Consistently, marked alternation in gene expression was detected with over 2000 differentially expressed genes associated with SGS pathogenesis (**Figure 1D**). These findings suggest that LTR from SGS patients are transcriptionally distinct from healthy controls.

**Table 2:** Patient demographics and characteristics.

Patient ID	Sex	Age	SGS status	Pathology	Recurrence	Surgical treatment	Cohort
4	Female	51	No	VFP	No	Yes	Test
7	Female	66	No	Leukoplakia	No	Yes	Test
8	Female	73	No	RRP	No	Yes	Test
11	Female	62	No	VFP	No	Yes	Test
14	Female	56	No	VFP	No	Yes	Test
2	Female	57	Yes	GPA	Yes	Yes	Test
6	Female	53	Yes	idiopathic SGS	Yes	Yes	Test
10	Female	72	Yes	idiopathic SGS	Yes	Yes	Test
13	Female	50	Yes	GPA	Yes	Yes	Test
21	Female	50	Yes	idiopathic SGS	Yes	Yes	Test
24	Female	53	Yes	Prior tubation	Yes	Yes	Test
25	Female	66	Yes	Tracheostomy	Yes	Yes	Test
26	Female	57	Yes	idiopathic SGS	Yes	Yes	Test
1	Female	87	No	VFD	No	Yes	Validation
3	Female	60	No	RRP	No	Yes	Validation
5	Female	64	No	RRP	No	Yes	Validation
12	Female	72	No	VFD	No	Yes	Validation
15	Female	75	No	VFD/RRP	No	Yes	Validation
17	Female	56	Yes	Tracheostomy	Yes	Yes	Validation
19	Female	66	Yes	idiopathic SGS	Yes	Yes	Validation
22	Female	44	Yes	idiopathic SGS	Yes	Yes	Validation

### Subglottic Stenosis Patients Exhibit Gene Signatures of Acute Inflammation

**Figure 2:**
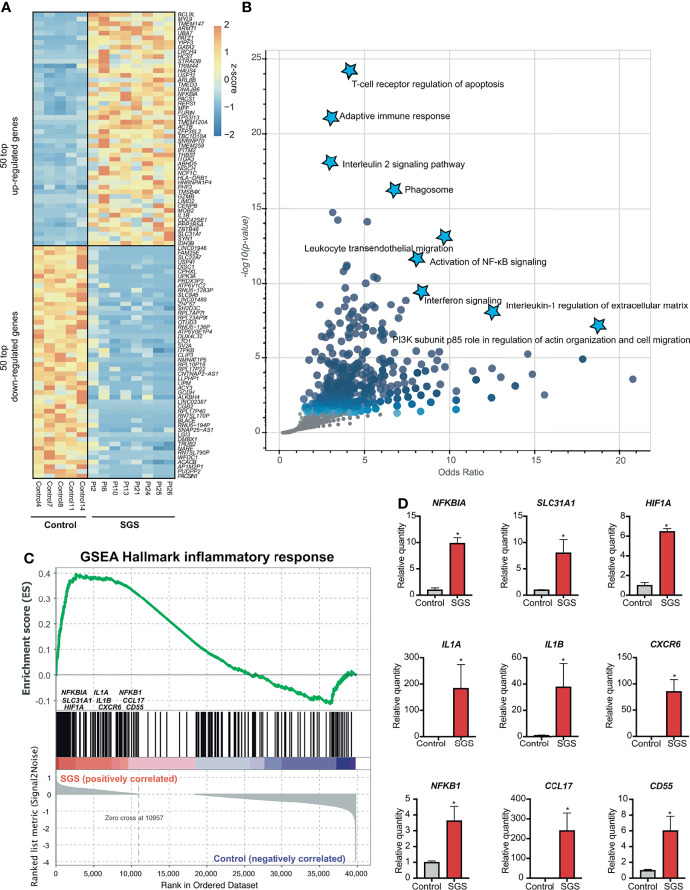
Subglottic stenosis patients exhibit gene signatures of acute inflammation. **(A)** Heatmap showing the top 50 upregulated genes and 50 down-regulated genes (ranked by FDR) comparing SGS patients to healthy controls. **(B)** Scatter plot showing gene ontology analysis of pathways enriched from up-regulated genes in SGS patients. **(C)** Gene set enrichment analysis (GSEA) for the human transcriptomes of healthy controls versus SGS patients showing enrichment of hallmark inflammatory response genes that were more upregulated in SGS patients. **(D)** Relative gene expression levels of *NFKBIA*, *SLC31A1*, *HIF1A*, *IL1A*, *IL1B*, *CXCR6*, *NFKB1*, *CCL17*, *CD55* as measured by qRT-PCR. Statistical tests were performed with Student’s t-test. *p < 0.05.

Of the over 2000 differentially expressed genes, SGS is associated with the upregulation of 967 genes and down-regulation of 1834 genes (**Figures 1D** and **2A**). Interestingly, gene ontology analysis revealed that the upregulated genes exhibited a highly pro-inflammatory profile, including T-cell receptor regulation of apoptosis, phagosome, interleukin, interferon, and NF-κB signaling pathways (**Figure 2B**). To better characterize the functionality of the differentially expressed genes, we performed geneset enrichment analysis (GSEA) with a pre-curated set of 200 genes previously implicated in inflammation. The result showed significant enrichment for genes belonging to the hallmark inflammatory responses in SGS, namely *NFKBIA*, *SLC31A1*, *HIF1A*, *CXCR6*, *CCL17*, *CD55*, *CD14*, *IL1A*, *IL1B*, *IL15*, *CXCL6*, *CXCL8*, *TGFBI*, and *NFKB1/RELA* (**Figure 2C**). Quantitative PCR of several of these targets in an independent cohort of SGS patients confirmed RNA-seq results that LTR from SGS patients exhibits gene expression signature of acute widespread inflammation (**Figure 2D**).

### Subglottic Stenosis Is Associated With Hyper-Activation of NF-κB and Its Downstream Inflammasome

**Figure 3:**
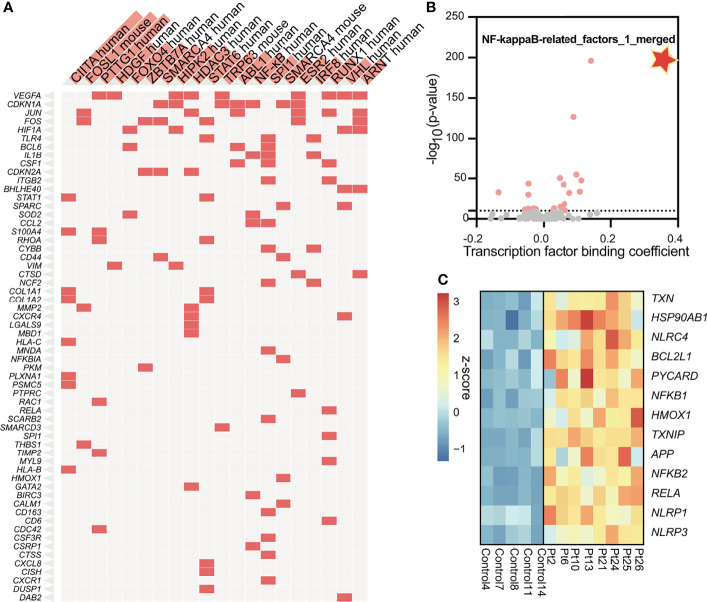
Subglottic stenosis is associated with hyper-activation of NF-kB and its downstream inflammasome. **(A)** Clustergram showing results from enrichment of transcription factors and their regulons from up-regulated genes in SGS patients using TRRUST Transcription Factors 2019 database. **(B)** Machine learning-based transcription factor binding analysis (TBA) applied to genomic regions 100kb upstream of up-regulated genes in SGS patients. DNA motifs are ranked by binding coefficient and significance of the motifs based on likelihood ratio test. **(C)** Heatmap showing expression levels of genes comprising the NF-κB-mediated inflammasome of healthy controls and SGS patients.

To understand the molecular mechanisms by which inflammation occurs in SGS, we applied transcription factor prediction analysis to genomic regions 100kb upstream of pro-inflammatory genes upregulated in SGS. DNA-sequence of these regions were highly enriched for motifs of NF-κB, FOS, STAT6, NCOR2, and RUNX1, all of which were previously implicated in inflammatory regulation (**Figure 3A**) (22, 23). Interestingly, NF-κB was also predicted as the most statistically significant positive transcription factor whose regulon was also coregulated by an independent machine-learning algorithm (**Figure 3B**). Indeed, multiple components of the NF-κB-mediated inflammasome (24) were also overexpressed in the setting of SGS (**Figure 3C**). Given that regulation of the inflammasome involves multiple “fail-safe” mechanisms that ensure proper resolution of inflammation (25), the hyperactivation of NF-κB and its downstream inflammatory mediators suggests a potential breach in one of the feedback mechanisms.

### Murine Model of Subglottic Stenosis Transcriptionally Captures Temporal Resolution of Airway Injury

**Figure 4:**
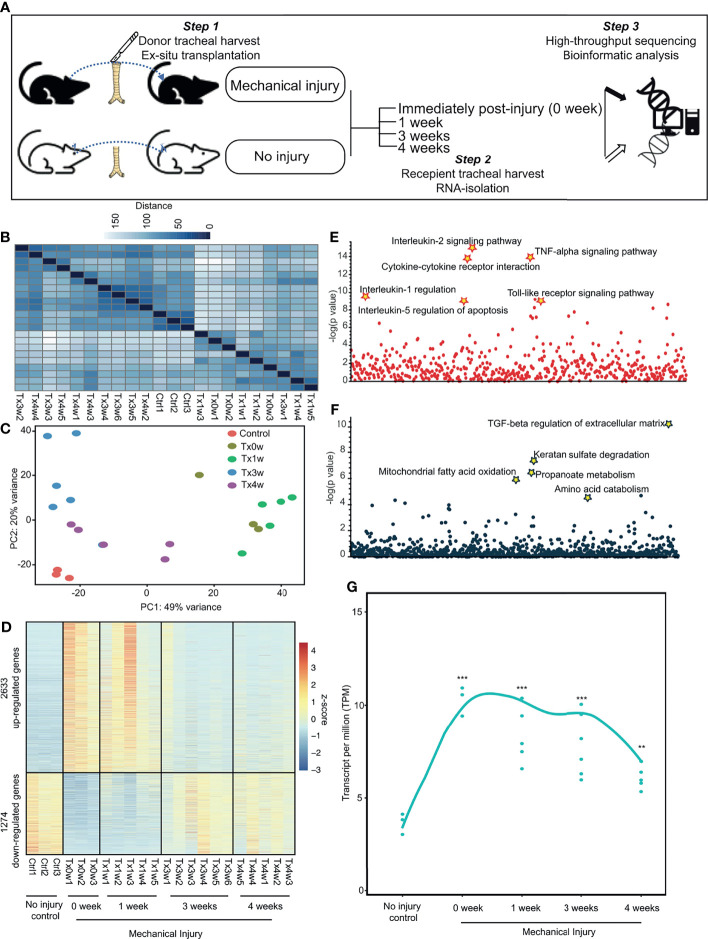
Murine model of subglottic stenosis transcriptionally captures temporal resolution of airway injury. **(A)** Schematics of the experimental design for ex-situ murine trachea harvest with subsequent injury, explantation, and collection at specific time points followed by transcriptomic profiling with RNA-sequencing. **(B)** Heatmap of sample-to-sample distances using the variance-stabilizing transformed values from RNA-seq experiments in murine tracheas from control (uninjured), or injured at 0-(immediately after), 1-, 3-, 4-weeks post-injury. **(C)** PCA plot using the variance-stabilizing transformed values from RNA-seq experiments in murine tracheas from control (uninjured), or injured at 0-(immediately after), 1-, 3-, 4-weeks post-injury. **(D)** Heatmap showing differentially expressed genes of murine tracheas from control (uninjured), or injured at 0-(immediately after), 1-, 3-, 4-weeks post-injury. **(E)** Manhattan plot displaying all enriched gene ontology pathways as points from up-regulated genes as identified in **(D)**. **(F)** Manhattan plot displaying all enriched gene ontology pathways as points from down-regulated genes as identified in **(D)**. **(G)** Average expression of differentially expressed genes as a smooth function of time. Statistical tests were performed with Mann-Whitney U-test. **p < 0.01, ***p < 0.001.

To gain more insights into the mechanisms of sustained inflammation in SGS, we utilized a previously developed SGS murine model (9) to assess the temporal evolution and resolution of inflammation resulting from direct laryngotracheal injury (**Figures 4A** and **S2**). Transcriptomic profiling of uninjured control tracheas, as well as injured tracheas at 0 week (immediately after), 1 week, 3 weeks, or 4 weeks after mechanical abrasion of the luminal cavity reveal that gene expression profiles from tracheas 0-1 weeks post-injury are more similar than those from 3-4 weeks of surgery, all of which were distinctive from uninjured controls (**Figures 4B, C**). Numerous genes were drastically upregulated in the acute phase 0-1 week following injury that were subsequently subdued at 3 and 4-week time points (**Figure 4D**). Conversely, there were a significant number of genes that were downregulated early on but substantially increased later. These marked global changes strongly reflect the robust yet dynamic transcriptional response to laryngotracheal injury. As expected, the majority of genes that were elevated shortly after injury function as pro-inflammatory activators, including cytokine-chemokine production and signaling (**Figure 4E**). By contrasts, early-suppressed genes that were induced later on involve in wound healing processes such as TGFβ regulation of extracellular matrix (**Figure 4F**). It is worth noting that, while the majority of transcriptional perturbation occurred immediately post-injury (0-week and 1-week), at 3-4 weeks after injury where injured tracheas developed tissue granulation histologically similar to human SGS (9), active inflammation and wound healing processes have not reached the level prior to injury (**Figure 4G**), suggesting that the development of SGS could be attributable to the absence of homeostasis between inflammation and wound healing.

### Human Subglottic Stenosis Resembles Acute Inflammation in Mice With Direct Laryngotracheal Injury

**Figure 5:**
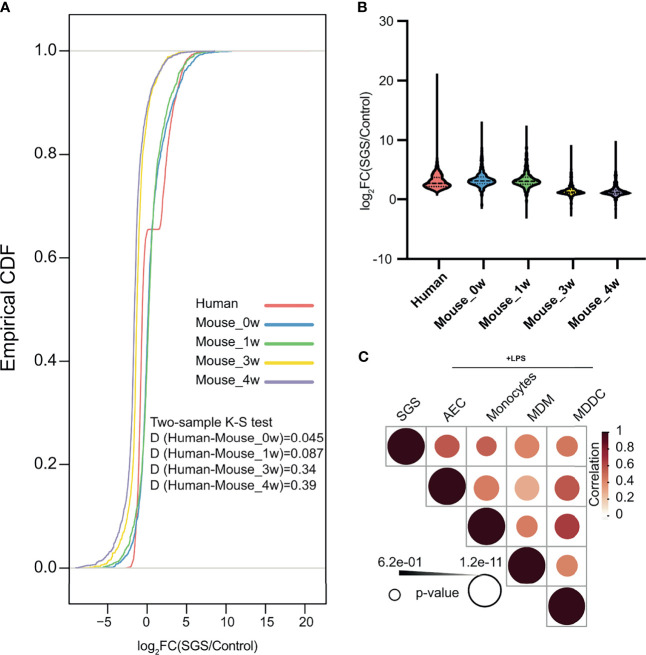
Human subglottic stenosis resembles acute inflammation in mice with direct laryngotracheal injury. **(A)** Empirical cumulative distribution function (CDF) of differentially expressed genes in SGS patients as compared to murine tracheas after 0-, 1-, 3-, or 4-weeks post-injury. Two-sided Kolmogorov–Smirnov test D statistics were performed for corresponding pairwise comparisons to compare the magnitude of the difference in distribution. All statistics shown with associated *p* < 0.05. **(B)** Violin plots showing differentially expressed up-regulated genes in SGS patients as compared to murine tracheas after 0-, 1-, 3-, or 4-weeks post-injury. **(C)** Heatmap showing correlation of gene expression among mucosal swab from SGS patients, and LPS-stimulated samples of airway epithelial cells (AEC), monocyte, monocyte-derived macrophages (MDM), and monocyte-deprived dendritic cells (MDCC).

To better delineate the chronology of inflammatory manifestations in SGS, we performed comparative genomics between human and mouse laryngotracheal transcriptomes using cumulative distribution functions to assess global shifts in gene expression of previously identified differentially expressed genes from our human dataset. Surprisingly, despite being classically associated with overwhelming fibrosis, transcriptional profiles of mucosal swabs from SGS patients bore stronger resemblance to those of acutely inflamed murine tracheas at 0 – 1 week post-injury, as indicated by a smaller Kolmogorov–Smirnov D-statistics, than those undergoing fibrotic changes at 3 – 4 weeks, exemplified by a larger Kolmogorov–Smirnov D statistics (**Figure 5A**). In fact, the degree of transcriptional activation of pro-inflammatory genes in human is much higher than that of mice, even immediately after mechanical injury (**Figure 5B**). To further characterize the inflammatory nature of SGS airway, we also compared our dataset to publicly available transcriptomes of human airway epithelium and different populations of monocytes exposed to lipopolysaccharide (LPS) (26, 27). Indeed, the inflammatory signature of SGS were strongly correlated with airway epithelial and immune cells post-LPS exposure (**Figure 5C**). This unexpected finding supports the notion that uncontrolled, recurrent, acute inflammation rather than abnormal fibrotic changes that may account for the luminal narrowing the airways of SGS patients.

### Persistent Acute Inflammation of Subglottic Stenosis Is Associated With Down-Regulation of Nitric Oxide Synthesis

**Figure 6:**
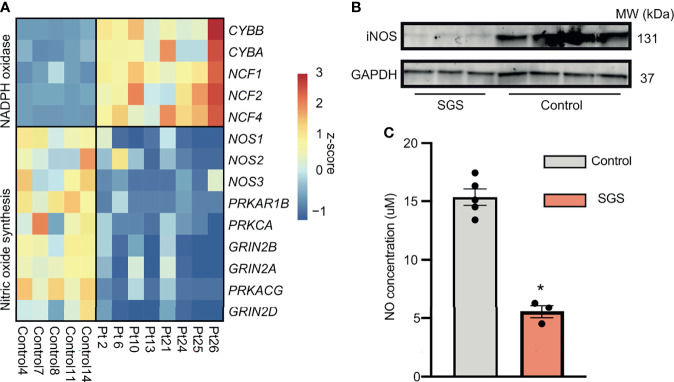
Persistent acute inflammation of subglottic stenosis is associated with down-regulation of nitric oxide synthesis. **(A)** Heatmap showing expression of genes component of either the NADPH oxidase or nitric oxide synthesis pathways from healthy controls and SGS patients. **(B)** Western blot of iNOS protein levels in laryngotracheal swabs of SGS patients (*n* = 3) and healthy controls (*n* = 4), with GADPH used as loading control. **(C)** Bar graph showing nitric oxide levels in laryngotracheal swabs of SGS patients and healthy controls. Statistical test was performed with Student’s t-test. *p < 0.05.

Since inflammation is a heavily-regulated, complex, immuno-protective mechanism underlying many pathological processes, we hypothesized that in order for SGS patients to maintain their highly inflamed airways, either immunomodulators that promote inflammation must be overexpressed or those that quench inflammation must be suppressed. An example of such dysregulation is the pathogenesis of chronic granulomatous disease notable for defects in reactive oxygen species (ROS) production in reactive oxygen species (ROS) production, which serve to both promote phagocytic destruction of microbes and attenuate production of proinflammatory cytokines (28, 29). We next examined major ROS generating pathways including NADPH oxidases and nitric oxide (NO) synthesis (30). Even though components of the NADPH oxidases were expectedly increased, those of NO signaling pathway were paradoxically down-regulated in SGS patients (**Figure 6A**). Furthermore, protein level of inducible nitric oxide synthetase (iNOS) was also lower in an independent cohort of SGS patients (**Figure 6B**), supporting the transcriptomic finding of decreased *NOS2* gene expression. In accordance with these findings, NO levels were significantly depleted in SGS mucosal swabs compared to controls (**Figure 6C**). Given that NO has been heavily implicated in immunoregulation of multiple inflammatory conditions (31–35), impairment in nitric oxide synthesis may be responsible for the recurrent hyper-inflamed LTR with subsequent luminal narrowing observed in SGS. These findings also warrant further investigations into to the clinical utility of targeting nitric oxide pathway pharmaceutically.

## Discussion

In this study we demonstrated, to the best of our knowledge, for the first time at a whole-genome level that hyperinflammation is the predominant feature of the SGS airway. Surprisingly, even though most of our patients have suffered from SGS for years, and on average at least 3 months out from either extubation or prior surgery, their airways are more consistent with an acute inflammatory response. Such persistent inflammatory response several months removed from the initial irritation provides strong supporting evidence that SGS airways are entrapped in the initial inflammatory phase of wound healing. These findings are consistent with a prior study demonstrating significant infiltration of neutrophils in scar tissues from SGS patients (10).

Dysfunctional immunoregulation has been implicated in pathological processes, from cancer, to metabolic and cardiovascular diseases. However, our data suggests that SGS airways resemble a particular state of immunodeficiency similar to that of chronic granulomatous disease (CGD), which is characterized by long-lasting production of pro-inflammatory cytokines and inflammatory manifestations coupled with persistent neutrophilic infiltration and granulomatous formation secondary to defects in ROS generation (36). In fact, not only X-linked female carriers of CGD were shown to have a higher incidence of autoimmune diseases (37), SGS patients are also almost exclusively female. ROS are not only crucial for phagocytic killing of ingested microbes (38), but also implicated as important signaling molecules in various immune processes, including providing negative feedback on Toll-like receptor activation and promoting anti-inflammatory properties (39–41). Interestingly, SGS airways were previously shown to be heavily colonized with unique members of the Moraxellaceae family (42), suggesting that a combination of pathological airway microbiome and insufficient microbial clearance might underscore the development of SGS. Furthermore, deficiency in nitric oxide synthesis and ROS production may also interfere with downregulation of NF-κB and its downstream mediators (43), further exacerbating the inflammatory responses, similarly reported in the development of diabetes, neuropathy, and cancer (44).

Although previous studies have provided isolated evidence of potential key immunomodulators of SGS, our transcriptional profiles of both human and mice SGS provide an atlas of all inflammatory mediators, including cytokines, chemokines, and growth factors that could serve as key biomarkers for SGS. Furthermore, we also provided the first proof of concept that mucosal swabs could offer valuable genetic information that could stratify diseased from healthy airways. Additionally, better understanding of the pathophysiology of SGS offers several options for future treatments. For example, our findings would support the anecdotal use of antibiotics prophylaxis as well as intralesional steroid injection (14). Interferon-gamma is being also used to enhance microbial clearance (45), while methods to increase levels of nitric oxide, such as inhaled NO, are also pharmacologically available (46).

Taken together, our study substantially expands on previous findings of abnormal wound healing in SGS patients by providing unbiased genomic evidence that their airways remain in persistent state of early inflammatory phase. Even though further validation and testing are required, our translational findings from a unique combination of both human tissue and mouse model provide multiple avenues for development of novel therapeutic, diagnostic, and prognostic options for SGS. This work adds to the growing knowledge of SGS that it is predominantly more a disease of inflammation than obstruction and moves us a step closer to making SGS a medically treated disease rather than a surgical one.

## Data Availability Statement

The datasets presented in this study can be found in online repositories. The names of the repository/repositories and accession number(s) can be found below: https://www.ncbi.nlm.nih.gov/geo/, GSE189336.

## Ethics Statement

The studies involving human participants were reviewed and approved by Hospital of University of Pennsylvania. The patients/participants provided their written informed consent to participate in this study. The animal study was reviewed and approved by Corporal Michael J. Crescenz Philadelphia Veterans Affairs Medical Center Institutional Animal Care and Use Committee.

## Author Contributions

HN, NC, and NM designed research. HN performed research and bioinformatics analysis. TC and NM collected human samples. HN and NM wrote the paper. All authors contributed to the article and approved the submitted version.

## Conflict of Interest

The authors declare that the research was conducted in the absence of any commercial or financial relationships that could be construed as a potential conflict of interest.

## Publisher’s Note

All claims expressed in this article are solely those of the authors and do not necessarily represent those of their affiliated organizations, or those of the publisher, the editors and the reviewers. Any product that may be evaluated in this article, or claim that may be made by its manufacturer, is not guaranteed or endorsed by the publisher.

## Supplementary Material

Supplementary Figure 1 Sequencing quality control. **(A)** Traces of RNA extracted from mucosal swabs of Control and SGS with associated RNA integrity number (RIN) as determined by Agilent 2100 bioanalyzer. **(B)** Traces of cDNA library size distribution from Control and SGS samples as determined by TapeStation. **(C)** Per base sequencing quality averaged for all Control and SGS samples. **(D)** Mapping statistics showing percentage of mapped versus unmapped reads to the human reference genome (hg38) in Control and SGS samples.

Supplementary Figure 2 Murine model of mechanical injury of trachea *ex-vivo* and subsequent transplantation.
