# Supplementary figures and images for: Persistent Inflammation and Nitric Oxide Dysregulation Are Transcriptomic Blueprints of Subglottic Stenosis
Source: Front Immunol. 2021 Dec 20;12:748533. doi: 10.3389/fimmu.2021.748533 (PMC8720859; doi:10.3389/fimmu.2021.748533)

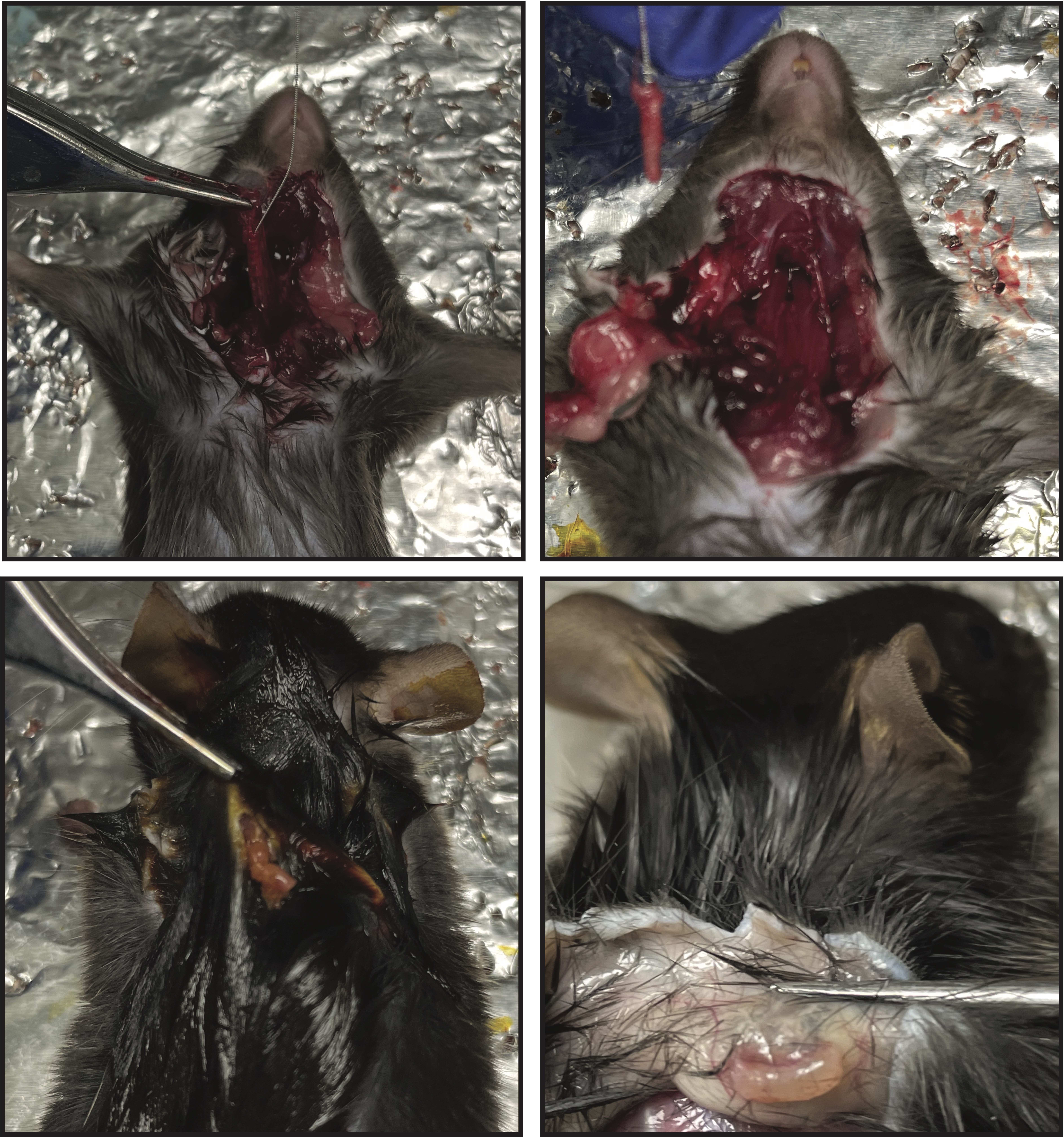

Supplement: Supplementary Figure 2 — Murine model of mechanical injury of trachea ex-vivo and subsequent transplantation. [file Image_2.jpeg]
